# Nitrate pollution in the Warta River (Poland) between 1958 and 2016: trends and causes

**DOI:** 10.1007/s11356-017-9798-3

**Published:** 2017-08-12

**Authors:** Józef Górski, Krzysztof Dragon, Piotr Michał Jan Kaczmarek

**Affiliations:** Institute of Geology, Department of Hydrogeology and Water Protection, Adam Mickiewicz University in Poznań, ul. Bogumiła Krygowskiego 12, 61-680 Poznan, Poland

**Keywords:** Nitrate contamination, Groundwater and surface water interaction, River water contamination, Warta River, Poland

## Abstract

The article presents analyses of long-term water quality data from the Warta River between 1958 and 2016. A clear increasing trend in nitrate concentrations was observed from 1958 to the early 1990s. This trend was mainly related to the increasing use of fertilizers in Poland in this period. Then, after the early 1990s, a slow decreasing trend related to improvements in water and sewage management and more rational fertilizer use was observed after political and economic changes in Poland. The influence of long-term hydrological droughts on nitrate concentrations was also investigated. Sharp increases in the nitrate concentration in surface water were related to the accumulation of contaminants in the soil and aeration zone during drought periods and the subsequent transport of these contaminants to groundwater and surface water via recharge infiltration after each drought period. The presented results highlight the importance of surface water–groundwater interactions and suggest that groundwater protection in an entire catchment area is essential for surface water quality protection.

## Introduction

The contamination of water by nitrates is a common phenomenon observed in many countries around the world (e.g., Hudak 2000; Rodvang and Simpkins 2001, Zurek et al. 2010; Lasagna et al. 2015). Nitrate contamination affects both surface water and groundwater (Chen et al. 2005; Hu et al. 2016; Oeurng et al. 2016). Because nitrates in drinking water can cause significant harm to human health, the European Union and the World Health Organization have determined the maximum permissible concentration of nitrates in drinking water to be 50 mg NO_3_/l (11.3 mg N–NO_3_/l) (Drinking Water Directive 98/83/EC 1998; WHO 2004). A similar permissible limit was also established in Poland (Rozporządzenie MZ 2015). To protect water from nitrate pollution across Europe, limitations have been implemented regarding the application of nitrogen to land, including in designated zones containing vulnerable aquifers (Nitrates Directive 91/676/EEC 1991).

The main source of nitrates in water is the application of both synthetic and organic fertilizers in agricultural areas (Harter et al. 2002; Dragon 2013). These fertilizers are non-point sources of pollution. The major point sources of nitrate pollution are septic tanks. These tanks are mainly located in rural settlement areas where no central sewer systems exist. These sources cause the contamination of both surface water and groundwater (Lasagna et al. 2016). Wastewater treatment effluent, which is usually discharged to ditches or rivers, is the largest pollution threat to surface water in urban areas with central sewage systems. In some specific situations when polluted surface water infiltrates into groundwater, groundwater contamination can also occur (Dragon et al. 2016).

It has often been assumed that surface water contamination by nitrates cause eutrophication. This process is especially important in lakes due to the relatively long water residence times (Hutchins 2012). Moreover, nitrate loading in rivers can cause eutrophication in coastal waters (Ruehl et al. 2007).

In Polish lowland areas, groundwater is a significant component of river discharge. It has been estimated that the groundwater contribution to rivers accounts for approximately 60% of river flows and even up to 80% in some catchments (Jokiel 1994). These values indicate that the quality of surface water can be largely affected by groundwater and that long-term analyses of surface water quality can be used in the evaluation of changes in groundwater quality. In the case of riverbank filtration (RBF) sites, surface water is the main source of potable water (Hiscock and Grischek 2002; Ray et al. 2002; Ray and Shamrukh Ed. 2009). At some RBF sites, surface water may account for 90% of the total water balance in wells (Górski and Przybyłek 2006). In this case, surface water quality has a considerable influence on the overall potable water quality.

The aim of this article is to evaluate nitrate contamination in the Warta River. This investigation was performed based on long-term river water quality data from 1958 to 2016. The influence of groundwater and climatic conditions on surface water quality was also investigated.

### Characterization of the Warta catchment area

**Fig. 1:**
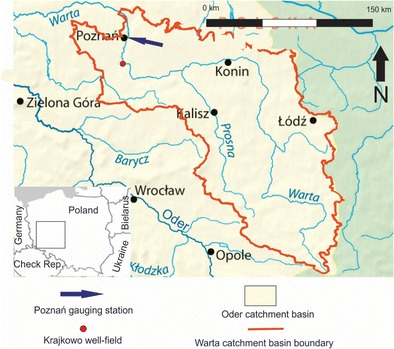
The map of the Warta catchment area

The southern area of the Warta River catchment, which is located in western Poland, was selected as the study area. The selected part of the catchment includes all the Warta tributaries located downstream of the Poznań gauging station (Fig. 1). The total area of the selected part of the catchment is 25,083 km^2^ and includes the upper and middle sections of the Warta catchment basin. The topographic conditions of this river system are characterized by two main factors: postglacial relief and the general northward slope of the land surface. In general, in the southern part of the catchment, the Warta River flows to the north. Then, the river turns west in the lowland area of the Warsaw-Berlin ice-marginal valley in the middle section of the catchment (Fig. 1).

**Fig. 2:**
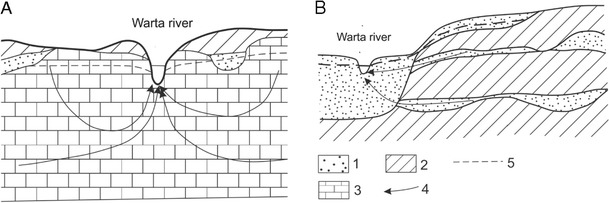
Hydrogeological characterization of the Warta river catchment area. **a** Upper part of the catchment. **b** Middle part of the catchment. Explanations: *1*—sands and gravels; *2*—limestones; *3*—glacial tills; *4*—flow lines; *5*—ground water table

In the upper part of the catchment, the aquifers consist of Jurassic and partly Upper Cretaceous carbonate formations. These rocks are locally overlain by thin layers of glacial tills and sands (Fig. 2a). The Jurassic rocks form karst aquifers. Thus, the shallow groundwater in this part of the catchment is critically vulnerable to pollution by nitrates. In the middle part of the catchment, aquifers can be found in Quaternary, Paleogene, and Neogene formations. The recharge of the Warta River in this part of the catchment mainly occurs via Quaternary aquifers. These recharge areas are mainly located in valleys, buried valleys, and outwash plains (Fig. 2b). Notably, the contamination of shallow groundwater by nitrates has been documented in the study area (Gorski 1989).

The mean annual precipitation in the Warta catchment basin, based on data from 1951 to 1980, is 561 mm. The annual precipitation in the driest year (1959) was only 329 mm and in the wettest year was 719 mm (Pasławski 1992). The highest precipitation occurs in the southwestern part of the catchment in the highland area (average annual precipitation varies from 607 to 618 mm). In the northern part of the catchment, the average annual precipitation is between 530 and 560 mm. The mean flow rate of the Warta River measured at the Poznań gauging station is 102 m^3^/s, and the mean low flow rate is 37 m^3^/s.

The total discharge from the catchment is estimated to be 3.57 l/s/km^2^, and groundwater discharge to the river is estimated to be 2.34 l/s/km^2^ (Pasławski and Koczorowska 1974). Additionally, the groundwater discharge is highly variable. The highest values of 3.0–4.5 l/s/km^2^ were documented in the upper part of the catchment, which has a substratum of karstic carbonate rocks. In the remainder of the catchment, the groundwater discharge is low and ranges between 1.5 and 2.0 l/s/km^2^. The groundwater discharge constitutes 65% of the mean river flow rate in the catchment and 85 to 93% of the mean river flow rate in the upper catchment. The groundwater discharge in the southern part of the catchment occurs via fractured and karstic carbonate rocks. In other parts of the catchment, the main sources of river water recharge are the shallow groundwater circulation systems, which are connected with the sands of river valleys and outwash plains, as well as shallow intertill aquifers (Fig. 2).

### Land use patterns and urbanization

The study area includes four cities with populations greater than 100,000 inhabitants and several dozen towns with populations greater than 15,000 inhabitants. In these cities and towns, sewage systems are connected to a central sewage treatment station. The treated effluent is usually discharged into the rivers. Villages and towns with populations less than 2000 inhabitants usually do not have central sewage systems. Domestic sewage is typically deposited in individual septic tanks, which are often incorrectly constructed and poorly preserved or used. This situation can lead to the leakage of untreated liquid waste into the ground or groundwater. The central sewage systems in the study area were modernized, and sewage treatment technologies have been modified over the last two decades. Prior to this period, a simple mechanical treatment technology was generally used. Over the past two decades, mechanical, biological, and chemical treatment technologies have been applied; thus, sewage treatment is more effective, and biogens are removed from effluent. Many new sewage systems have been constructed over the past two decades and the percentage of inhabitants using central sewage systems increased from 40% in 1995 to 70% at present (XII 2016) (Dmowska Ed. 2014). Land use outside residential regions is dominated by agricultural activities. Chemical fertilizers and the spreading of manure are potential sources of groundwater contamination.

## Materials and methods

To investigate the changes in the water quality of the Warta River over time, long-term monitoring data from 1958 to 2016 were used. The Warta River analyses were performed in Aquanet SA (Poznań waterworks operator) as part of the local monitoring system of the Debina well field, where the managed aquifer recharge method is used. The frequency of sampling during the investigation period was a minimum of one sample per month. Meteorological data were obtained from the Poznań Lawica meteorological gauging station, which is maintained by the Institute of Meteorology and Water Management—National Research Institute (IMGW-PIB). For groundwater quality characterization, data were obtained from the monitoring system of the Mosina–Krajkowo well field, which supplies Poznań city. The groundwater sampling was performed at this site during and after the hydrological drought.

## Results and discussion

**Fig. 3:**
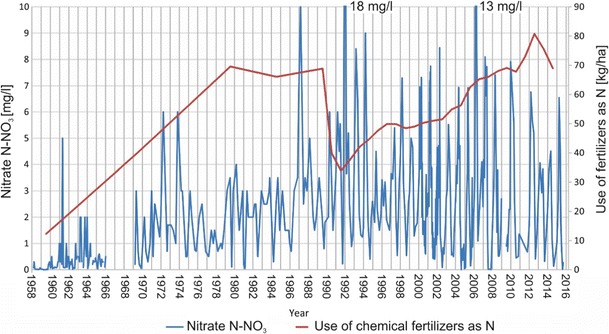
The long-term nitrate concentration changes in Warta River water and the use of nitrogen fertilizers in Poland

Figure 3 presents the changes in nitrate concentrations (expressed as N–NO_3_) in the Warta River from 1958 to 2016. Nitrate–nitrogen concentrations are highly variable and range from 0 to 18 mg N–NO_3_/l. The interpretation of the changes in nitrate concentrations over the study period is quite difficult due to long-term changes, seasonal changes, and various trends in meteorological conditions (drought and wet seasons).

**Fig. 4:**
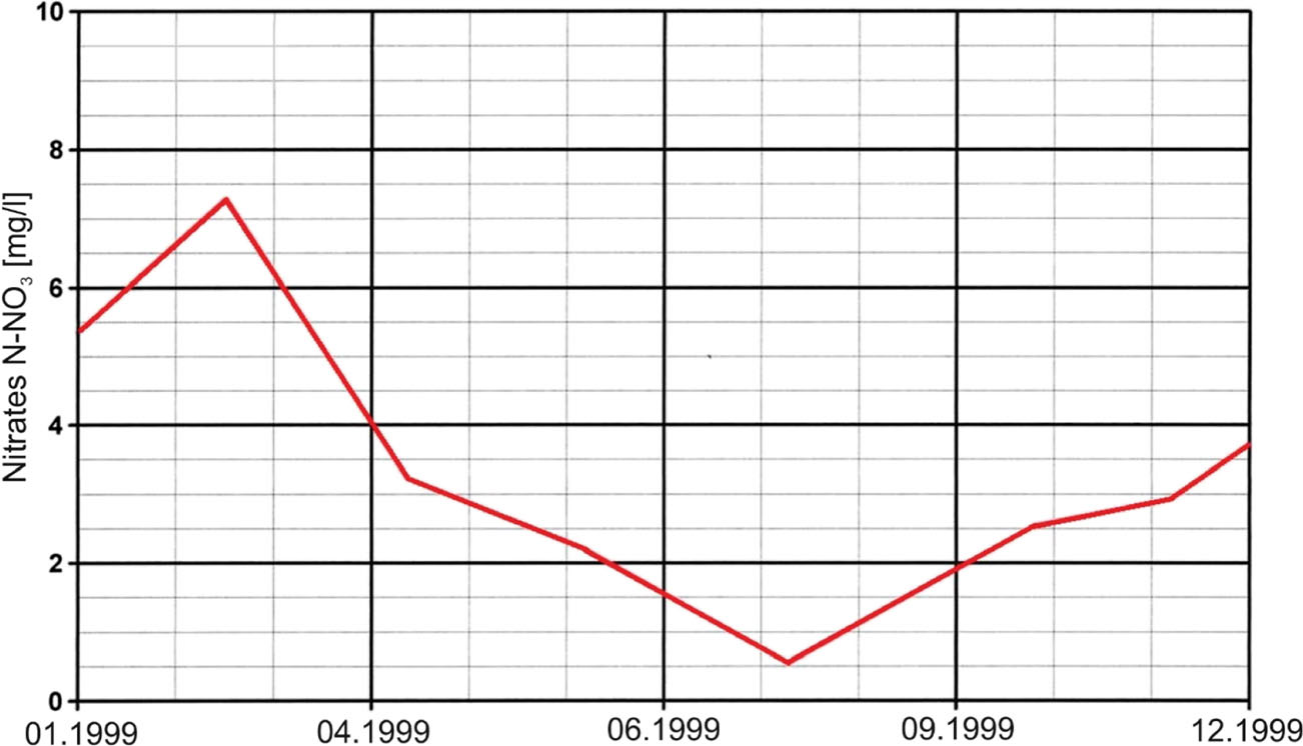
Typical seasonal changes in nitrate concentrations in Warta River water (chosen year 1999)

Seasonal variations in nitrate concentrations are related to the phases of flora and fauna growth in the river water, which result from seasonal temperature changes and are a major factor in regulating the biological processes that determine N cycling (Howden and Burt 2008). Figure 4 shows the typical seasonal changes in nitrate concentrations during a chosen year (1999). High nitrate concentrations usually occur in autumn, winter, and early spring when the temperature is low and biological activity is limited, whereas in late spring and summer, the nitrate concentration is usually low because of the vegetative period and the biological absorption of nitrates that occurs during this period.

**Fig. 5:**
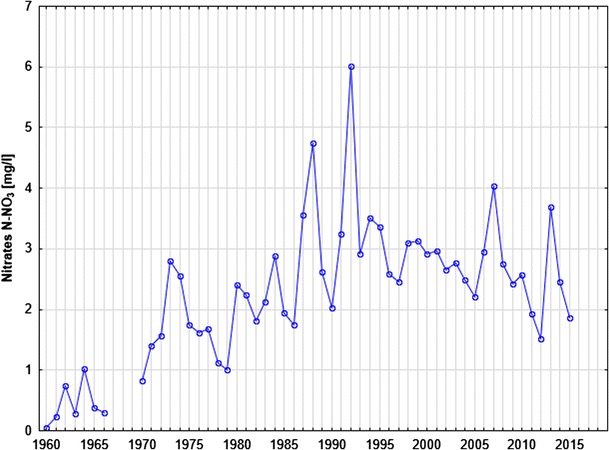
The average annual concentration of nitrates in Warta River water

**Fig. 6:**
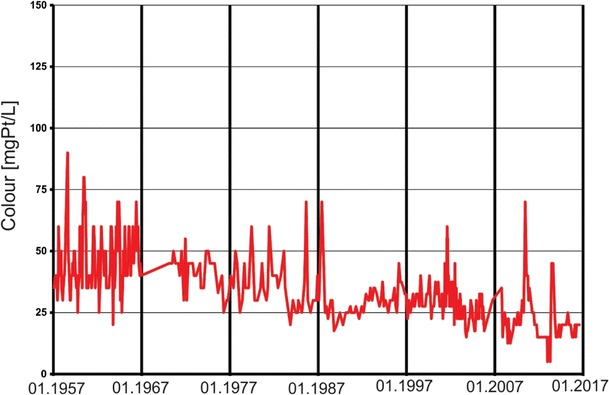
Changes in the Warta River water colour

**Fig. 7:**
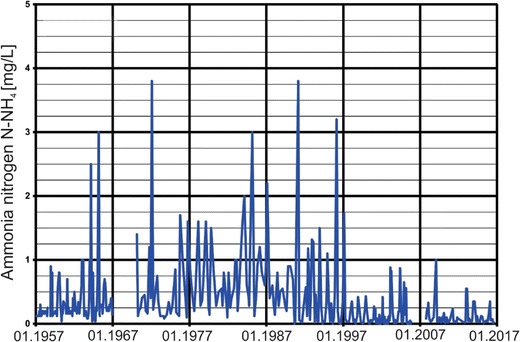
Changes in ammoniacal nitrogen concentration in Warta River water

Despite the clear seasonal variations in nitrate concentrations, long-term changes are visible in Fig. 3. These trends are also indicated on the plot of the average annual nitrate concentrations (Fig. 5). An increasing trend in nitrate concentrations is visible from the start of the observation period (1958) to the early 1990s. After the early 1990s, a slow decreasing trend with decreasing minimum concentrations and low maximal peaks can be observed. These trends are related to two main factors: the use of fertilizers and wastewater management. From the 1958s to the 1980s, the use of fertilizers increased from 10 to 70 kg/ha (Fig. 3). After the 1990s, a sharp decrease in fertilizer use occurred due to political and economic changes in Poland after 1989. The increasing trend in nitrate concentrations until the 1990s and the decreasing trend after this period clearly correspond with the use of fertilizers in Poland. These trends demonstrate that agricultural activities are the main factor responsible for nitrate concentrations in river water. Despite the increased use of fertilizers, the observed decreasing trend in nitrate concentrations after the 1990s is the result of more rational fertilizer use after economic changes occurred in Poland. Additionally, the implementation of the Nitrate Directive (91/676/EEC 1991) affected this trend. The second factor related to this decreasing trend is the improvement in sewage treatment technology that occurred in Poland over the past two decades. Such improvements include the construction of many new sewage systems and the limitation of wastewater discharge directly into surface waters based on political and economic changes in Poland. The improvement of sewage treatment technology also caused a decrease in ammonia nitrogen concentrations and an improvement in the color of river water (Figs. 6 and 7). The associated nitrate changes suggest that both factors (agriculture and wastewater) are responsible for variations in nitrate concentration levels in surface water, but the nitrate concentration peak in the 1990s suggests that agriculture is the dominant factor.

The observed trends in nitrate concentrations in river water are similar to those observed in other parts of Europe in regions where agriculture is the dominant type of land use. For example, Roberts and Marsh (1987) observed significant and rapid increases in nitrate concentrations in rivers in southern and eastern England in the 1960s resulting from postwar agricultural intensification. Using long-term data, Howden and Burt (2008) reported a highly significant positive correlation between the annual mean nitrate concentration and the concentration of N–NO_3_ from 1978 to 2007 in the Frome and Piddle catchments in Dorset (UK). They also found that correlations between the nitrate concentration and climatic variables (e.g., rainfall) were weak and rarely significant. However, climate variability is considered an important driver of the hydrological cycle (Oeurng et al. 2016). Notably, flood events are particularly important because they transport nutrients from different sources in the upstream catchment to the river (Buda and DeWalle 2009).

**Fig. 8:**
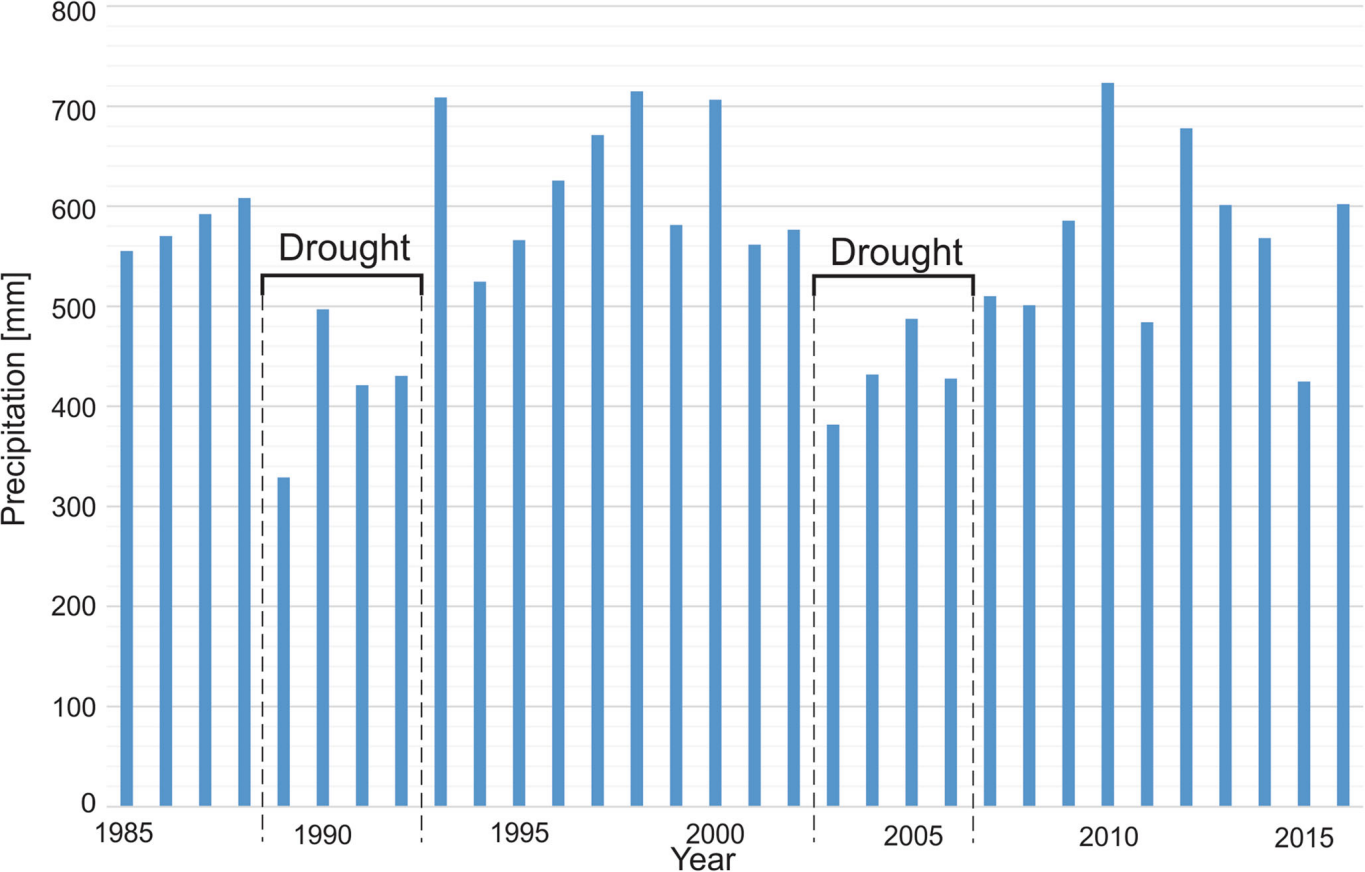
The average annual precipitation in the Warta River catchment basin (Poznan–Lawica meteorological gauging station)

**Fig. 9:**
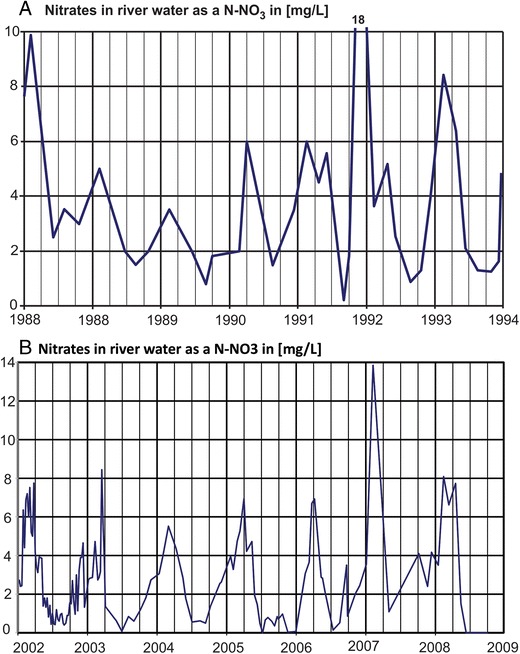
Changes in nitrate concentrations in Warta River water during hydrological droughts (**a** hydrologic drought between 1989 and 1992; **b** hydrologic drought between 2003 and 2006)

In Fig. 8, which shows the annual precipitation in the Warta River catchment region from 1985 to 2016, two long periods of hydrological drought can be observed. The first dry period spanned from 1989 to the end of 1992 (until the end of summer). After this period, a very rainy year occurred (1993). The second dry period occurred between 2003 and 2006. For clarity, the influences of these events on the nitrate behavior in the river water and changes in the nitrate concentration are presented at a detailed scale (Fig. 9). Periods of hydrological drought are typically characterized by low nitrate concentrations in river water. In both dry periods, the changes in the seasonal concentration can be observed but are constrained to a relatively small concentration range (between 1 and 6 mg N–NO_3_/l). Notably, at the end of the hydrological drought periods, sudden increases in nitrate concentrations occurred, and maximal peak concentrations were reached. In 1992, the nitrate concentration was as high as 18 mg N–NO_3_/l (Fig. 9a), and in 2006, it was 14 mg N–NO_3_/l (Fig. 9b). A statistical analysis of the nitrate concentration is presented in Table 1. Seasonal nitrate fluctuations are related to the biological activity discussed above. The main source of nitrates in river water during drought periods is wastewater. The influence of agriculture is limited because low precipitation results in relatively little recharge infiltration, and the agricultural contamination load accumulates in the soil and aeration zone. Following drought, the contamination load is activated and transported to shallow groundwater and then to surface water. Characteristically, the contamination load is activated immediately after the first increase in precipitation that occurs after a drought period due to the influence of drainage systems (Dragon et al. 2016). This phenomenon was confirmed by a study of nitrates in groundwater conducted based on a network of eight production and observation wells around an unconfined aquifer in the Warsaw–Berlin ice-marginal valley (Table 2). These wells are located in the Mosina–Krajkowo well field, which is very close to the Warta River. The nitrate concentrations during drought were low at all monitoring sites (0.02–0.4 mg N–NO_3_/l). Then, 3 months after the end of the dry period, a significant increase in nitrate concentrations was observed. At the end of 1992, the concentration increased at all monitoring sites to a range of 1.2–6.0 mg N–NO_3_/l. One year later, the nitrate concentrations decreased to previous levels. These results confirm the phenomenon of nitrate accumulation in agricultural areas during drought and explain their high concentrations in river water after the end of hydrological droughts. Notably, the analyzed wells are located within the protected area of the well field where the contamination influence is limited. In agricultural areas, especially those with shallow groundwater tables, the increase in the nitrate concentration will likely be high.

**Table 1:** The statistical characterization of nitrate concentration

Indicated period	Number	Minimum	Average	Median	Maximum	Standard deviation
Whole data set	458	0	2.2	1.7	18.0	2.18
Hydrological drought 1989–1992	16	0.22	2.79	2.0	6.0	1.83
Hydrological drought 2003–2006	63	0	2.53	2.56	8.44	1.98

**Table 2:** Nitrate concentration in groundwater during and after the drought

Well number	Depth of well screen [m b.s.]	Nitrates as a N–NO_3_ (mg/l)		
		August 1989 (during drought)	December 1992 (3 months after drought)	September 1993 (1 year after drought)
2	33.3–46.8	0.02	1.2	0.025
36	24.3–37.8	0.1	1.6	0.03
X	7.5–12.5	0.02	1.2	0
8b	17.0–19.0	0.3	2	0
18b/2	12.0–14.0	0.2	1.4	0.02
47b/1	17.0–19.0	0.04	6	1
71b	26.2–30.9	0.4	1.4	0.1
6k	7.2–9.2	0.1	1.6	0.015

## Conclusions

At the beginning of the data collection period (1958), a clear increase was observed in the nitrate concentrations in the Warta River. This trend changed after the early 1990s, when a general decrease in nitrate concentrations occurred. The nitrate concentration trends are strongly correlated with the use of fertilizers in Poland. Fertilizer use sharply declined in the early 1990s due to political and economic changes in Poland. The increase in fertilizer use after 2000 did not cause nitrate concentrations to increase due to more rational application methods. Additionally, nitrate trends are linked to sewage wastewater. The modernization of sewage treatment technology and the construction of new sewage systems after the 1990s caused a decrease in nitrate concentrations in river water. The decreased influence of sewage wastewater on river water quality is also supported by a systematic decrease in ammonia nitrogen concentrations and the improved color of river water, which has been noted since the 1990s.

Climate changes cause characteristic fluctuations in nitrate concentrations in river water. During drought periods, nitrate fluctuations are constrained to a relatively small range of concentrations. However, high characteristic peaks in nitrate concentrations were observed after two hydrological droughts (1989–1992 and 2003–2006). These peaks are related to the enrichment of nitrates in groundwater after the accumulation of contaminants in the soil and aeration zone during drought and the transport of contamination plumes via recharging infiltration immediately after the dry season.

The presented results illustrate the importance of surface water–groundwater interactions. Groundwater protection in the catchment area is needed to maintain and improve the surface water quality. Such protection is important, especially in the case of surface water used for water supply purposes, as well as at riverbank filtration sites where surface water is the main component of the total water balance.
